# Composite structure of human mind revealed by HuPoTest

**DOI:** 10.1192/j.eurpsy.2023.2197

**Published:** 2023-07-19

**Authors:** G. Dragan

**Affiliations:** GDF Databanks, Bucuresti, Romania

## Abstract

**Introduction:**

HuPoTest is a mental test and training procedure as well discovered incidentally in 1967 during developing color photos in complete dark in successive baths for seconds. I observed that I was able to count seconds mentally with high repeatability. I was curious to check my skill by comparing my count with a commercial stopwatch with the help of another person. The results were very interesting, so I extending progressively this experiment to other persons and I was able to establish the correlation between more and more calculated parameters with the particular psychic patterns of the persons under test (PUT). In fact, HuPoTest is a calibration of personal mental-timer compared to a standard stopwatch. I test myself periodically and the results are published (Dragan, GDF Databanks Bull., 2021; 25(6), 1-4).

**Objectives:**

Study of a large variety and number of systems in transformation revealed their composite structure, namely these have one component in transformation (Ctr) and an inert one (Cin) (Dragan, GDF Databanks Bull., 2011; 15(2), 1-19).This is the case of the human mind which is in permanent transformation, i.e. in continuous more or less coherent thinking. HuPoTest can establish the size of Ctr, Cin, ctr – the size of kinetic unit constituting Ctr and the coupling strength (CS) between Ctr and Cin.

**Methods:**

PUT has to count mentally 5, 10, 15 and 20 seconds for 8-10 times each value by using a standard stopwatch and comparing the statistically retrieved matrix of measured values with the above mentioned imposed standard values. I was able to test face-to-face approximately 4000 persons during more than 50 years by collecting a huge databank. I explained in many publications the exact procedure, the majority of calculations and the significance of the resulted parameters by correlating the obtained results with the PUT mental pattern (Dragan, GDF Databanks Bull., 2019; 23(1) 1-6).

**Results:**

The final results obtained by myself in the period of 28.05 – 03.07.2022 (A to CII) with overall of 48 tests are presented in attached figures by using a standard stopwatch created by Mihai Dornea developer at National Instruments on LabView® platform with an accuracy of 10 μs. Four distinct behaviors are revealed showing the good evolution of mind by decreasing Ctr and CS and increasing ctr by correct and continuous training.

**Image:**

**Figure:**
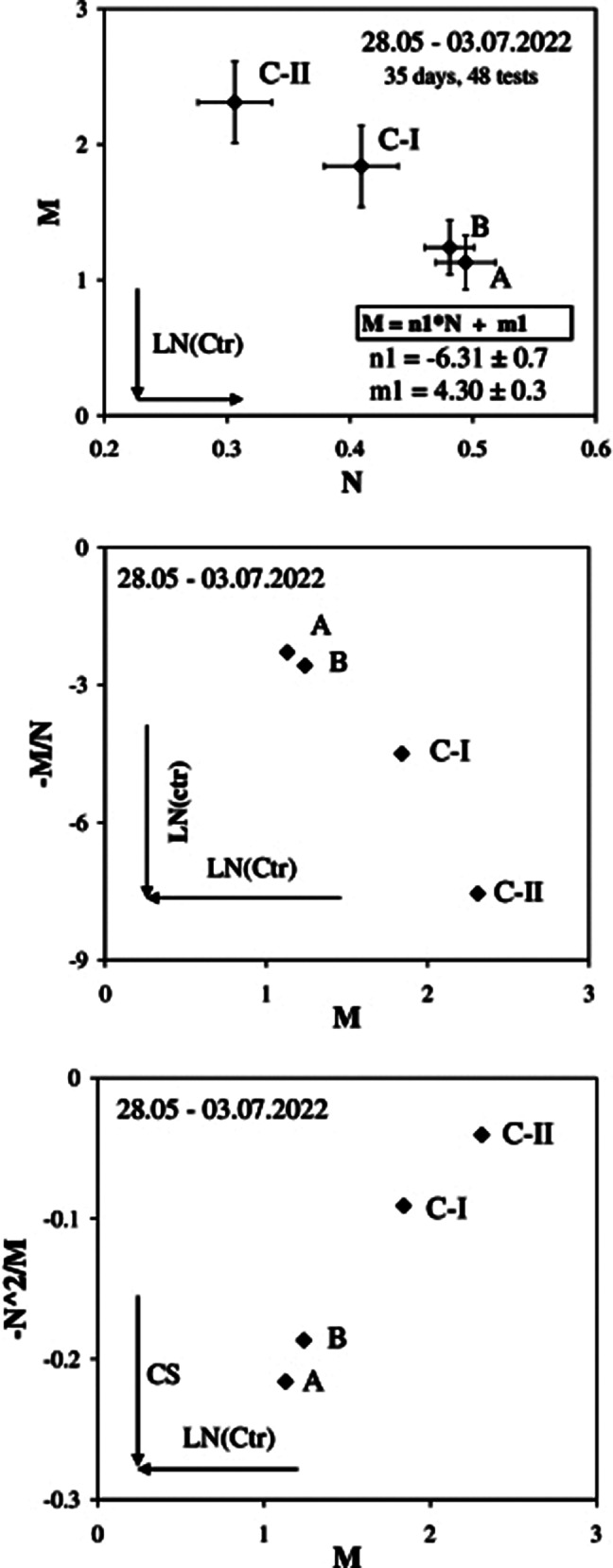

**Conclusions:**

HuPoTest is a highly efficient test and mental training procedure as well applied periodically in view to achieve a good and stable mental performance ensuring also a stable and good body health according to the well known quotations “sound mind in sound body” and "mind is the builder and body is the temple”. Such mental training becomes absolutely necessary taking into account that the survivors of the already started global war culminating at around 2035 will be persons with properly trained minds. I am available without any obligation for help anyone willing to try and practice HuPoTest.

**Disclosure of Interest:**

None Declared

